# Medical Students' and Trainees' Country-By-Gender Profiles: Hofstede's Cultural Dimensions Across Sixteen Diverse Countries

**DOI:** 10.3389/fmed.2021.746288

**Published:** 2022-02-08

**Authors:** Lynn V. Monrouxe, Madawa Chandratilake, Julie Chen, Shakuntala Chhabra, Lingbing Zheng, Patrício S. Costa, Young-Mee Lee, Orit Karnieli-Miller, Hiroshi Nishigori, Kathryn Ogden, Teresa Pawlikowska, Arnoldo Riquelme, Ahsan Sethi, Diantha Soemantri, Andy Wearn, Liz Wolvaardt, Muhamad Saiful Bahri Yusoff, Sze-Yuen Yau

**Affiliations:** ^1^Faculty of Medicine and Health, The University of Sydney, NSW, Australia; ^2^Chang Gung Medical Education Research Centre, Chang Gung Memorial Hospital, Linkou, Taiwan; ^3^Faculty of Medicine, University of Kelaniya, Ragama, Sri Lanka; ^4^Department of Family Medicine and Primary Care, Bau Institute of Medical and Health Sciences Education, The University of Hong Kong, Hong Kong, Hong Kong SAR, China; ^5^Department of Obstetrics and Gynaecology, Mahatma Gandhi Institute of Medical Sciences, Wardha, India; ^6^Department of Education, Peking University Health Science Center, Beijing, China; ^7^Life and Health Sciences Research Institute (ICVS), School of Medicine, University of Minho, Braga, Portugal; ^8^ICVS/3B's, PT Government Associate Laboratory, Largo do Paço, Portugal; ^9^Faculty of Psychology and Education Sciences, University of Porto, Porto, Portugal; ^10^Department of Medical Education, Korea University College of Medicine, Seoul, South Korea; ^11^Department of Medical Education, Sackler Faculty of Medicine, Tel Aviv University, Tel Aviv, Israel; ^12^Center for Medical Education, Nagoya University Graduate School of Medicine, Nagoya, Japan; ^13^Tasmanian School of Medicine, University of Tasmania, Hobart, Tasmania, TAS, Australia; ^14^Health Professions Education Centre, RCSI University of Medicine and Health Sciences, Dublin, Ireland; ^15^Department of Gastroenterology, Centre for Medical Education and Health Sciences, Department of Health Sciences, Faculty of Medicine, Pontificia Universidad Católica de Chile, Santiago, Chile; ^16^Department of Public Health, College of Health Sciences, QU Health, Qatar University, Doha, Qatar; ^17^Department of Medical Education, Faculty of Medicine Universitas Indonesia, Jakarta, Indonesia; ^18^Medical Education Center, Faculty of Medicine, Indonesian Medical Education and Research Institute, Jakarta, Indonesia; ^19^Faculty of Medical and Health Sciences, University of Auckland, Auckland, New Zealand; ^20^School of Health Systems and Public Health, Faculty of Health Sciences, University of Pretoria, Pretoria, South Africa; ^21^Department of Medical Education, School of Medical Sciences, Universiti Sains Malaysia, Penang, Malaysia

**Keywords:** internationalization, culture, gender, medical students, medical trainees, uncertainty

## Abstract

**Purpose:**

The global mobility of medical student and trainee populations has drawn researchers' attention to consider internationalization in medical education. Recently, researchers have focused on cultural diversity, predominately drawing on Hofstede's cross-cultural analysis of cultural dimensions from general population data to explain their findings. However, to date no research has been specifically undertaken to examine cultural dimensions within a medical student or trainee population. This is problematic as within-country differences between gender and professional groups have been identified within these dimensions. We address this gap by drawing on the theoretical concept of national context effects: specifically Hofstede's six-dimensional perspective. In doing so we examine medical students' and trainees' country profiles across dimensions, country-by-gender clustering, and differences between our data and Hofstede's general population data.

**Methods:**

We undertook a cross-cultural online questionnaire study (eight languages) containing Hofstede's 2013 Values Survey. Our questionnaire was live between 1st March to 19th Aug 2018, and December 2018 to mitigate country holiday periods. We recruited undergraduate medical students and trainees with at least 6-months' clinical training using school-specific methods including emails, announcements, and snowballing.

**Results:**

We received 2,529 responses. Sixteen countries were retained for analyses (*n* = 2,307, 91%): Australia, Chile, China, Hong Kong, India, Indonesia, Ireland, Israel, Japan, Malaysia, New Zealand, Pakistan, South Africa, South Korea, Sri-Lanka, Taiwan. Power distance and masculinity are homogenous across countries. Uncertainty avoidance shows the greatest diversity. We identified four country clusters. Masculinity and uncertainty are uncorrelated with Hofstede's general population data.

**Conclusions:**

Our medical student and trainee data provides medical education researchers with more appropriate cultural dimension profiles than those from general population data. Country cluster profiles stimulate useful hypotheses for further research, especially as patterning between clusters cuts across traditional Eastern-Western divides with national culture being stronger than gendered influences. The Uncertainty dimension with its complex pattern across clusters is a particularly fruitful avenue for further investigation.

## Introduction

Medical students sometimes seek opportunities to study medicine abroad (1, 2): particularly students from Eastern countries studying in the West (3, 4). This has drawn researchers' attention to the issue of internationalization in medical education. The debate has historically been dominated by two contrasting perspectives: one emphasizing global education homogenization (5, 6), the other on cultural and contextual diversity (7, 8). Recently, researchers have focused on the cultural diversity issue, trying to make sense of their data by drawing on Hofstede's cultural framework (9–11). However, no research has specifically examined Hofstede's cultural dimensions within a medical student population. Rather, researchers draw upon data from general country profiles to explain their findings (10, 12, 13). This is problematic as within-country differences between professional groups and gender have been identified (9, 10, 14). We address this gap by uniquely describing medical students' and trainees' responses across 16 countries: developing country-by-gender cultural profiles to facilitate culturally attuned educational experiences internationally (15).

Cultures comprise unique sets of characteristics, beliefs, and social norms (9). Although current medical programs increasingly emphasize the importance of cultural safety and cross-cultural communication, the cultural complexity of the learning environment is often underrated (16, 17). For example, the direct import of Western education into Eastern cultures can result in students' professional development being impaired; with reports of problems relating to their progress, cultural integration, and other difficulties (e.g., hierarchy) (7).

The theoretical basis informing our cross-cultural study is underpinned by the concept of *national context effects* (18). This asserts that every national context exerts a specific and strong influence on those socialized within it (9, 10, 19), developing shared norms, beliefs, and behaviors of *in-group* members (10). National culture scores are not about individuals, nor about the justification for stereotyping individuals; rather they tell us something about national societies. This is akin to any other demographic characteristics (e.g., gender, sexuality) whereby research findings related to characteristics are about the group, not specific people.

Therefore, as we introduce our cross-cultural work derived from the field of cultural social psychology, it is important to note that this field (and our work) does not, and has never, asserted an essentialist viewpoint on culture. Neither is this our perspective or intention. Thus, work in this genre recognizes the existence of cross-cultural *similarities*, but also important cross-cultural *differences*. There is also a recognition of variability *within* different cultural groups, but considerations of mean differences between these groups is important. Therefore, comparisons of the central tendency (or the average) across different cultural groups are the focus of our work. As with all research that looks at demographic comparisons, at no point do we have any assumptions of demographic homogeneity. We also believe that culture changes over time with education, training, and influences between cultural communities (10). Furthermore, with globalization, individuals can be bicultural or multicultural. Given this, there are no assumptions of an individual's cultural background based on physical appearance.

Numerous cross-cultural theories have been suggested to explain how workplace values are influenced by national culture (9, 20–24). The most popular perspective comes from Hofstede (22) whose book, Culture's Consequences, is one of the 25 most-cited social science books (25, 26). Hofstede identified six national cultural dimensions (6-Ds) (27) describing the extent to which countries are similar. Box 1 outlines these dimensions, short names, and descriptions with reference to understanding high-low scores on each dimension and brief research on how each dimension relates to healthcare and educational contexts (10).

Box 1 **The six key cultural dimensions (with short names) as measured by Hofstede's 2013 values survey and related research.**

**Power distance [power]**

In any society, inequality exists: some people are more powerful than others, some have more status, some have more money, and so on. This situation is accepted in some cultures more than others. Power distance therefore refers to “*the extent to which less powerful members of institutions and organizations within a country expect and accept that power is distributed unequally*” [(10), p. 61].

***VSM2013***: High scores on this dimension suggest that hierarchical order is relatively uncontested in that culture, whereas low scores suggest the culture values equality and questions hierarchical assumptions.

***Implications:*** Cultures with high scores on Power Distance have been found to have large student-teacher dependencies whereby teachers are respected (even feared), initiating all communication with knowledge being considered the imparting of personal wisdom. Challenging a teacher is considered disrespectful. Cultures with low scores on Power Distance have been found to be more student-centric, students are expected to find their own intellectual paths, ask questions, and even disagree with teachers (10).

**Individualism vs. Collectivism [individualism]**

Some societies (probably the majority) value the interests of the group over that of the individual (Collectivism, the power of the group). In other societies, the interests of the individual prevail (Individualism).

***VSM2013***: High scores on this dimension suggests the culture values a loose-knit (Individualist) community, whereas low scores suggest the culture values tight-knit (Collectivist) communities.

***Implications:*** In Collectivist cultures, students tend to speak up only when sanctioned by their group, education is about learning things, and gaining entry into higher status groups. For Individualist cultures, students are expected to speak up, education is about learning how to learn, to increase economic worth and self-respect (10).

**Masculinity vs. Femininity [masculinity]**

A masculine society is one in which the emotional gender roles are distinct: males are tough and assertive, focusing on material wealth; females should be modest and tender, and value quality of life. A feminine society is one in which these traditional emotional gender roles merge and both males and females adopt the emotional female gender role.

***VSM2013***: High scores on this dimension suggest the culture is more assertive, status, and results-oriented with clear distinct emotional gender roles. Low scores suggest the culture is modest and warm, focusing on quality of life.

***Implications:*** In feminine cultures, educators prefer to openly praise students who are struggling, giving them encouragement, rather than openly praise those succeeding. Collaborative learning is valued. Openly striving for excellence and being competitive are considered a masculine virtue and ridiculed (modesty is preferred). By contrast, openly striving for and celebrating excellence and competitiveness are valued in a masculine culture: and in strong masculine cultures failure in school can lead to suicide whereas in a feminine culture it is considered a relatively minor event (9).

**Uncertainty avoidance [uncertainty]**

We all have to deal with the fact that we can't predict the future, working out ways of doing this. These ways of managing uncertainty are culturally mediated. Extremely high uncertainty creates tolerance anxiety. Technologies, laws, and religions are ways in which cultures influence uncertainty tolerance.

***VSM2013***: High scores on this dimension suggests the culture is avoidant of uncertainty with a greater fear of the unknown than lower-scoring cultures.

***Implications:*** Students in high uncertainty cultures tend to prefer a single correct answer situation (truth), and to be rewarded for accuracy. Those in uncertainty-tolerant cultures value open-ended, unstructured, learning situations with the possibility of multiple correct ways of achieving the “right” answer, accept a “don't know” answer form their teachers and being rewarded for originality (9, 10).

**Long term orientation vs. Short term orientation [orientation]**

This dimension relates to issues such as thrift, perseverance, adherence to traditions, face-saving, and ordering relationships by status. Thus, long-term orientation represents attitudes toward future rewards, especially perseverance and thrift. Short-term orientation is around fostering past-present virtues, especially tradition, face-saving, and the fulfillment of social obligations.

***VSM2013***: High scores on this dimension relates to long-term orientation values, with low scores relating to short-term orientation (as above).

***Implications:*** Thinking preferences differ between short-term orientated cultures. Cultures scoring high (long-term orientation) afford priority to common sense, employ a more synthetic approach to thinking and accept multiple and opposing “truths” (if X is true then the opposite of X can also be true). Cultures scoring low (short-term orientation) demonstrate a need for cognitive consistency, abstract rationality and analytical thinking are preferred, and if X is true then the opposite of X must be false (10).

**Indulgence vs. Restraint [indulgence]**

Indulgence refers to having relatively free gratification of our desires, with personal fun and enjoyment being key. Alternatively, restraint is the opposite to this, whereby personal gratification is curbed.

***VSM2013***: High scores on this dimension relates to the indulgence end of the dimension suggesting the culture values free gratification (enjoying life and having fun). Low scores relate to restraint, suggesting the culture reflects the regulation of gratification through strict social norms.

***Implications:*** High scoring cultures (indulgence) suggest the culture facilitates freedom of speech, and an easy structure governing relationships between educators and students. Low-scoring cultures (restraint) suggest a situation where students are subordinate relative to their educators, with low student motivation (28).

Hofstede's work, alongside many others who attempt to classify national culture, has been challenged in a number of ways including deeply philosophical issues (e.g., the ontological nature of this approach) alongside more procedural issues (e.g., low face validity of some questionnaire items). Each of these critiques have been addressed accordingly (see Venkateswaran and Ojha for a detailed analysis) (29). Despite limitations, Hofstede's framework remains the most practical, wide-ranging and robust quantitative account of culture. Compared with other models, Hofstede's data includes a larger sample of countries (22), fewer dimensions, with all dimensions being statistically distinct (23, 30). Additionally, Hofstede's dimensions are widely replicated with no loss of validity: a review of 180 empirical studies found Hofstede's framework to successfully predict cross-cultural differences across a range of constructs, including change management, decision-making, leadership, and group processes (30, 31).

Within medical education, research across a variety of topics (e.g., international medical graduates, curricula reform, professionalism, sexual harassment) has been undertaken to understand different country cultures and practices, drawing on Hofstede's perspective (13, 32–37). Through such knowledge we might better understand how a country's cultural aspects align or misalign with another's values and practices and how these might impact positively or negatively within university and hospital workplaces.

For example, at the level of the curricula, research drawing on Hofstede's model (specifically around power, uncertainty, and individualism) suggests that culture impacts on the implementation of best-practice educational models (13, 37). At the level of the topic, differences around medical professionalism have been linked to Hofstede's cultural dimensions (e.g., individualism) (36). Thus understanding how physicians from different cultures prioritize professional values and behaviors, and how these are transmitted to students is of central importance when we consider the mobility of the medical profession (2, 35, 36). Finally, at an individual level, cultural differences of incoming overseas-trained physicians to a country in comparison to home-trained physicians (specifically Hofstede's power, uncertainty, and individualism dimensions) have been identified (34). Thus, anticipating the effect of the potential impact of physicians' cultural experiences can enable the development of effective transition programmes (15).

Despite the plethora of work attempting to understand medical students', trainees', and doctors' cultural differences through Hofstede's dimensions, none have directly measured Hofstede's values. Rather, they draw on general country profiles developed though Hofstede's IBM data base of nationally collected data for comparison (10, 12). However, Hofstede argues that researchers should be mindful when extrapolating from whole-country data due to possible gender, social class, education levels, and occupational nuances within country scores (9, 10). Presently no research has investigated cultural similarities and differences with a large-scale, cross-cultural medical student/trainee population. We address this gap by examining patterns of national cultural dimensions that are present in this cohort across 16 countries. We present our analysis as the starting point from which medical education researchers examining cross-cultural issues can more meaningfully draw upon to understand medical education issues in the context of globalization and cultural sensitivity. Specifically, we answer the following research questions (RQs):

RQ1: How do medical students and trainees from different countries score on the cultural dimensions measured by Hofstede's Values Survey (VSM, 2013) and does this differ by gender?

RQ2: How do country-by-gender profiles cluster across cultural dimensions?

RQ3: To what extent are our medical student/trainee population similar to the same-country populations as measured by Hofstede?

## Methods

### Study Design

We undertook a cross-cultural, cross-sectional study, using an online questionnaire extending previous research (38). The new questionnaire examines associations between culture, professionalism dilemmas, compliance/resistance to those dilemmas, and moral distress of medical students. Demographic questions were included, comprising participants' gender (female, male, neutral/non-binary), age group (classified in 5-year intervals), nationality, and country of study. We also include questions around the university at which participants are studying, the degree, and number of years. We measure culture using Hofstede's VSM 2013. This article focuses on data collected in this section (27). The countries studied are a convenience sample based on existing collaborators' history of successful teamworking, alongside within-investigator snowballing.

### Hofstede's VSM 2013

The VSM 2013 (30-items) assesses culturally influenced values of countries (27). The 6-Ds are each measured by four questions. Except demography, responses are from “1” (utmost importance/strongly agree) to “5” (very little/no importance/strongly disagree) (9). We translated (and back-translated) the VSM 2013 items from English into Hebrew, Indonesian, Japanese, Korean, Mandarin-Simplified/Traditional, and Spanish.

### Sampling and Recruitment

Each study site obtained ethical approval. Our questionnaire was live between 1st March to 19th Aug 2018, and December 2018 to mitigate country holiday periods. We recruited undergraduate medical students and trainees with at least 6-months' clinical training using school-specific methods including: email, virtual learning environments, student noticeboards, social networking (e.g., YouTube, Facebook), messaging apps (e.g., Line), and snowballing via student organizations. Participation was voluntary, and responses anonymous. We could not ascertain actual response rates as we were unable to confirm total numbers invited. Further, for this analysis we are only interested in participants who are presently studying in their own country as culture is so pervasive that those currently studying overseas are likely to already be influenced by their new culture.

### Participants

Two thousand five hundred and twenty-nine responded across 51 countries (Supplementary Material 1) and 84 medical institutions (Supplementary Material 2). Listwise deletion excluded 222 cases (9%); of these *n* = 4 had no variation across VSM 2013 questions; *n* = 17 were studying overseas; and *n* = 201 were from countries with *n* < 20 responses (27). Sixteen countries (74 institutions) were retained for analyses (*n* = 2,307, 91%, Table 1): Australia, Chile, China, Hong Kong, India, Indonesia, Ireland, Israel, Japan, Malaysia, New Zealand, Pakistan, South Africa, South Korea, Sri-Lanka, Taiwan.

**Table 1:** Nationality of participants (*N* = 2,307).

	***n* (%) of responses**	***n*** **(and % of gender) for item responses**	
**Nationality**		**Female**	**Male**
Pakistani	429 (19%)	215 (50.1%)	212 (49.4%)
Indonesian	263 (11%)	181 (68.8%)	79 (30%)
Indian	256 (11%)	118 (46.1%)	137 (53.5%)
Chinese	210 (9%)	70 (33.3%)	135 (64.3%)
Taiwanese	192 (8%)	85 (44.3%)	103 (53.6%)
Malaysian	169 (7%)	130 (76.9%)	38 (22.5%)
Sri-Lankan	106 (5%)	74 (69.8%)	31 (29.2%)
Australian	101 (4%)	68 (67.3%)	32 (31.7%)
New Zealander	96 (4%)	59 (61.5%)	37 (38.5%)
South African	95 (4%)	67 (70.5%)	27 (28.4%)
Israeli	85 (4%)	60 (70.6%)	25 (29.4%)
Chilean	82 (4%)	47 (57.3%)	35 (42.7%)
South Korean	73 (3%)	45 (61.6%)	28 (38.4%)
Japanese	56 (2%)	21 (37.5%)	35 (62.5%)
Irish	55 (2%)	32 (58.2%)	23 (41.8%)
Hong Konger	39 (2%)	9 (23.1%)^*^	30 (76.9%)

* *These data comprise <20 sample size so were excluded from the hierarchical clustering analysis considering gender differences*.

### Statistical Analyses

Using SPSS (39), we analyzed the data according to the VSM 2013 manual (27). For cultural dimension mean scores, we computed mean scores for Pakistan (having the highest response number), adding constants (positive/negative) across our data, fitting all mean scores with Hofstede's base cultural data (Table 2) (12). We performed two cluster analyses, assessing the extent to which each country by gender (using mean scores) is similar to another by cultural dimension, and country profiles across dimensions: (1) a *k*-mean cluster classified countries into *high*-*medium*-*low* groups by cultural dimension; (2) an hierarchical clustering analysis with Ward's algorithm applied to squared Euclidean distances produced homogenous country groupings based on cultural dimension profiles. A follow-up one-way ANOVA investigated differences in cluster classifications. *k*-mean cluster analysis was applied, rather than the traditional median split or single cut-off point method. We did this to aggregate countries, as our participant sample was unbalanced, considering the differences between participants for each country, and the number of countries (*n* = 16) being relatively small. The median split approach can be affected by the skewness of the sample scores, with the *k*-mean cluster analysis being less affected. This facilitated countries being classified into the group most similar to their dimensions' profile. We conducted a correlation analysis to understand the relationship between our data (specific to a medical professional culture) with Hofstede's base data (representing the dominant local culture). For this, we ranked our data according to the countries appearing in Hofstede's database for all dimensions separately. We performed bivariate association analyses among ranks to investigate dimension similarities between data.

**Table 2:** Cultural dimensions' formulas.

**Original formulas:**
*Power* = 35 × (*m*07−*m*02) + 25 × (*m*20 − *m*23) + ∁(*pd*)
*Individualism* = 35 × (*m*04 − *m*01) + 35 × (*m*09 − *m*06) + ∁(*ic*)
*Masculinity* = 35 × (*m*05 − *m*03) + 35 × (*m*08 − *m*10) + ∁(*mf*)
*Uncertainty* = 40 × (*m*18 − *m*15) + 25 × (*m*21 − *m*24) + ∁(*ua*)
*Orientation* = 40 × (*m*13 − *m*14) + 25 × (*m*19 − *m*22) + ∁(*Is*)
*Indulgance* = 35 × (*m*012 − *m*011) + 40 × (*m*17 − *m*16) + ∁(*ir*)
**Formulas with adjusted constant:**
*Power* = 35 × (*m*07 − *m*02) + 25 × (*m*20 − *m*23) + 51
*Individualism* = 35 × (*m*04 − *m*01) + 35 × (*m*09 − *m*06) + 22
*Masculinity* = 35 × (*m*05 − *m*03) + 35 × (*m*08 − *m*10) + 41
*Uncertainty* = 40 × (*m*18 − *m*15) + 25 × (*m*21 − *m*24) + 98
*Orientation* = 40 × (*m*13 − *m*14) + 25 × (*m*19 − *m*22) + 71
*Indulgance* = 35 × (*m*012 − *m*011) + 40 × (*m*17 − *m*16) − 35

*m, mean (e.g., m07, mean score for question 07)*.

## Results

### Mean Score Distribution for Cultural Dimensions (RQ1)

**Figure 1:**
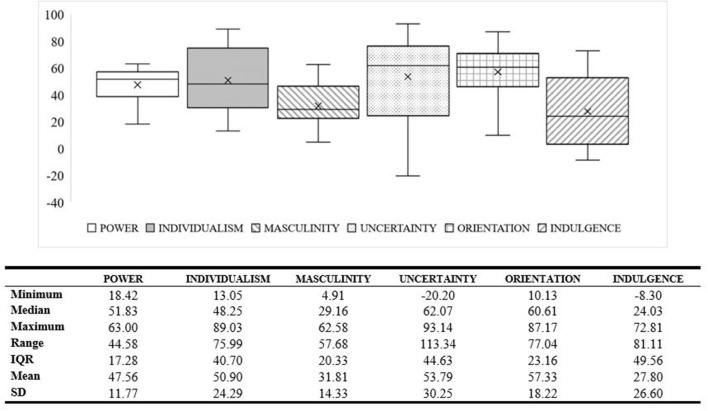
Distribution of country-by-gender scores in each dimension.

Most adjusted dimension scores are between 0 and 100, excepting: uncertainty for Israeli male respondents (uncertainty = −20) and Australian male respondents (uncertainty = −1); indulgence for Chinese male respondents (indulgence = −8), Japanese male respondents (indulgence = −4), and Pakistani female respondents (indulgence = −2: Figure 1). Power had the smallest mean difference range (*Range* = 45; *SD* = 12), with uncertainty having the greatest distribution (*Range* = 113; *SD* = 30).

**Figure 2:**
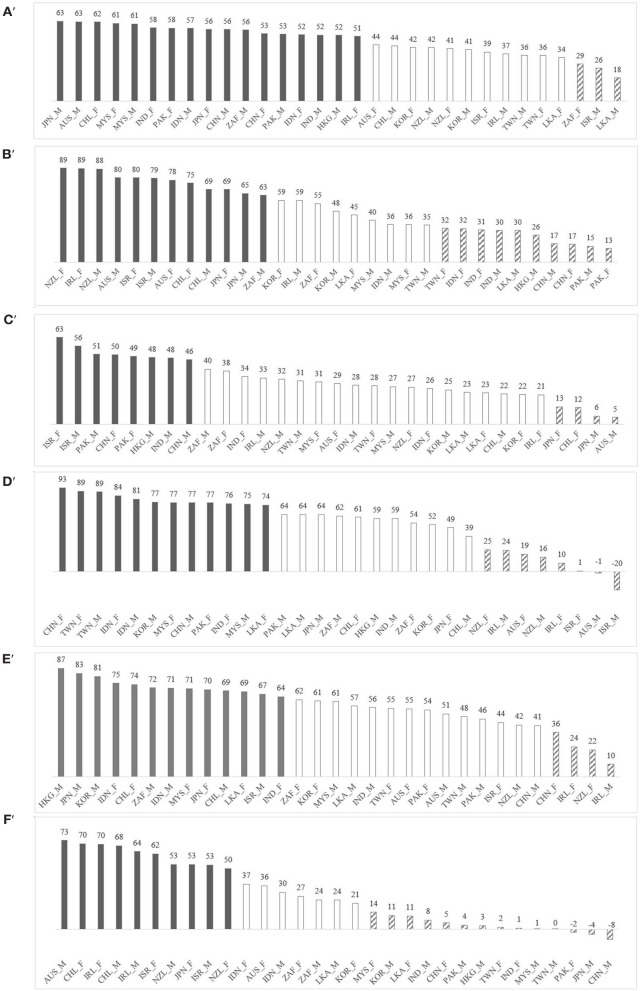
Country-by-gender ranking for Hofstede's six cultural dimensions: M, male; F, male. Groups were aggregate using *k*-mean cluster (*k* = 3): high (gray bars), medium (white bars), and low (diagonal-stripe bars). **(A)** power, **(B)** individualism, **(C)** masculinity, **(D)** uncertainty, **(E)** orientation, and **(F)** indulgence.

We divided each dimension into three categories (*low*-*medium*-*high* values), as country sample distributions are asymmetric with outliers. High and low scores on each dimension were described individually in Box 1, where medium scores were those that do not indicate a clear preference toward either extreme. *k*-mean cluster analysis revealed statistically significant differences between countries and gender (Figures 2A–F); power *F*_(228)_ = 114.9, *p* < 0.001; individualism *F*_(2, 28)_ = 98.4, *p* < 0.001; masculinity *F*_(2, 28)_ = 93.6, *p* < 0.001; uncertainty *F*_(2, 28)_ = 127.8, *p* < 0.001; orientation *F*_(2, 28)_ = 77.3, *p* < 0.001; and indulgence *F*_(2, 28)_ = 197.6, *p* < 0.001.

### Profiling Country-By-Gender Clusters (RQ2)

**Figure 3:**
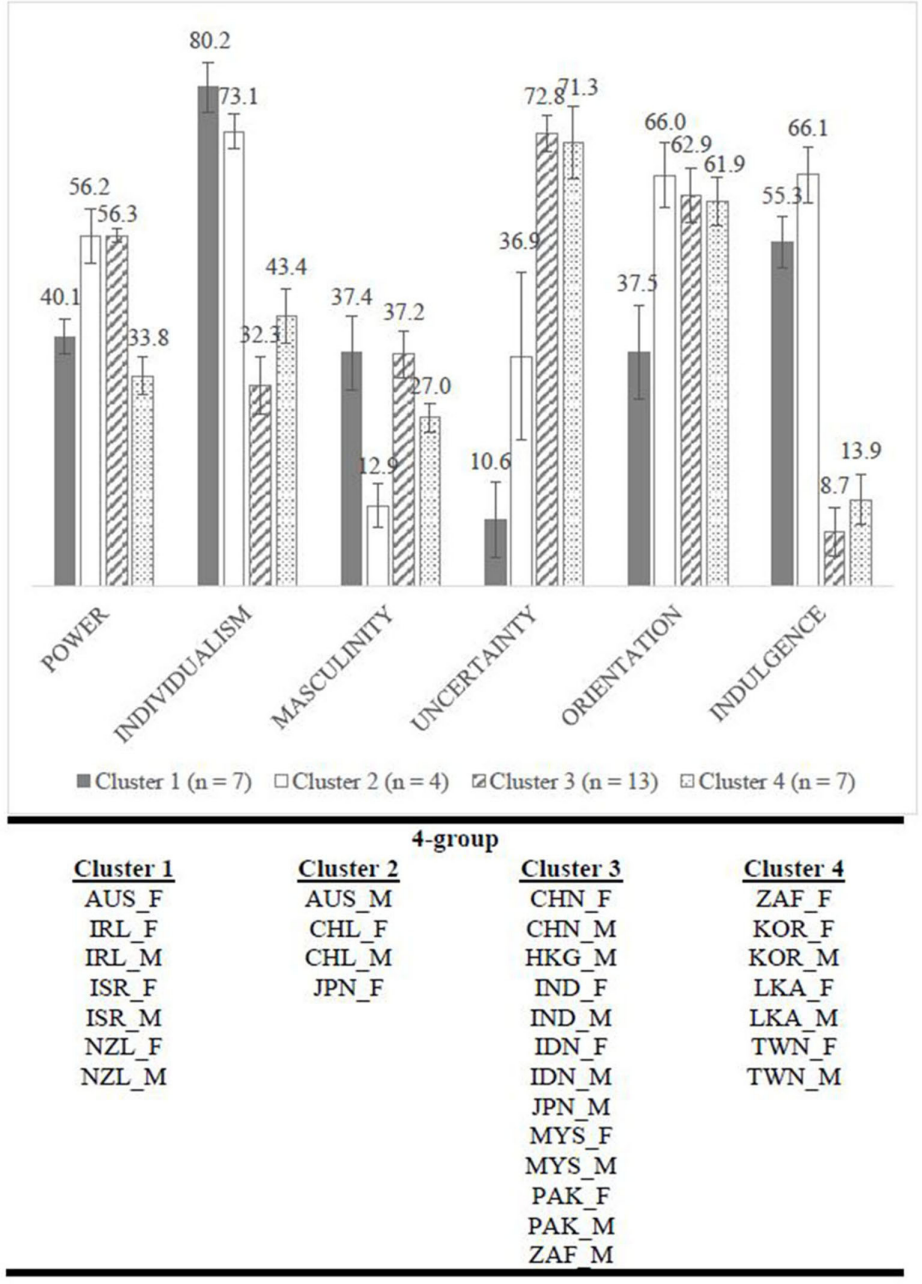
Overall pattern of clusters' characteristics based on Hofstede's six cultural dimensions by gender. Mean scores by cluster and error bar represented 95% CI.

Our hierarchical cluster analysis (16 country-by-gender) model, using statistical information and face validity, resulted in four distinctive and meaningful clusters accounting for 54% of variance (Figure 3): although a two-cluster model appeared an elegant solution, the within-cluster differences were large thus compromising its' utility (Supplementary Material 3). One-way ANOVA revealed statistically significant differences in cluster scores by dimension: power [*F*_(3, 27)_ = 23.5, *p* < 0.001, η^2^ = 0.72]; individualism [*F*_(3, 27)_ = 23.8, *p* < 0.001, η^2^ = 0.73]; masculinity *F*_(3, 27)_ = 4.97, *p* < 0.05, η^2^ = 0.36]; uncertainty [*F*_(3, 27)_ = 29.6, *p* < 0.001, η^2^ = 0.77]; orientation [*F*_(3, 27)_ = 5.11, *p* < 0.05, η^2^ = 0.36]; indulgence [*F*_(3, 27)_ = 39.3, *p* < 0.001, η^2^ = 0.81]. The effects of all 6-Ds are statistically significant (*p* < 0.05) with effect sizes being large (η^2^ > 0.14). However, power, individualism, uncertainty, and indulgence have greater effect sizes compared to masculinity and orientation. Over 70% of the total variance can be accounted for by power, individualism, uncertainty, and indulgence scores (η^2^ > 0.70). Comparatively, only 36% of the total variance can be accounted for by masculinity and orientation (η^2^ = 0.36). *Post-hoc* analysis (Tukey correction) indicates that Cluster 1 scores statistically significantly higher on masculinity than Cluster 2, but lower on power and orientation; Cluster 2 scores significantly higher on individualism than Cluster 3, but lower on masculinity, uncertainty, and indulgence; Cluster 3 scores significantly higher on power than Cluster 4, *p* < 0.05.

### Hofstede's Original Data Comparison (RQ3)

We undertook a correlation between our data (Supplementary Material 4) and Hofstede's base culture data (https://geerthofstede.com/research-and-vsm/dimension-data-matrix/ Accessed 11th January 2022). Differences range from 0.78 for indulgence to −0.004 for uncertainty (Table 3). Significant correlations are found for power, individualism, and indulgence, with large difference for masculinity and uncertainty. For example, Japan ranks first on masculinity in Hofstede's data, but last in ours; for uncertainty, Israel ranks fourth in Hofstede's general ranking but fourteenth in ours, with China ranking thirteenth in Hofstede's analysis but third in ours.

**Table 3:** Adjusted ranking of country by dimension for medical participants' data (MPR) compared with Hofstede's general ranking (GR).

	**Power**		**Individualism**		**Masculinity**		**Uncertainty**		**Orientation**		**Indulgence**	
**MPR**	**MPR**	**GR**	**MPR**	**GR**	**MPR**	**GR**	**MPR**	**GR**	**MPR**	**GR**	**MPR**	**GR**
Australia	9	11	3	1	12	4	13	7	9	15	4	2
Chile	7	6	5	9	13	14	10	2	4	13	1	3
China	3	2	13	10	3	3	3	13	13	4	14	12
Hong Kong	8	5	11	8	4	6	9	14	1	6	9	13
India	5	4	12	5	5	7	6	10	8	7	11	11
Indonesia	6	3	10	13	10	11	2	9	3	5	5	9
Ireland	10	12	4	3	9	2	12	12	15	14	2	4
Israel	14	14	2	4	1	10	14	4	12	10	–	n/d
Japan	2	10	6	6	14	1	8	1	2	2	7	8
Malaysia	1	1	8	7	7	8	4	11	6	9	10	6
New Zealand	12	13	1	2	8	5	11	8	14	12	3	1
Pakistan	4	9	14	14	2	9	5	5	11	8	13	14
South Africa	–	n/d	–	n/d	–	n/d	–	n/d	7	11	6	5
South Korea	11	7	7	11	11	13	7	3	5	1	8	10
Taiwan	13	8	9	12	6	12	1	6	10	2	12	7
Correlations between ranks	0.57^*^		0.71^*^		–0.03		–0.004		0.25		0.78^*^	

*Sri Lanka does not appear in Hofstede's general ranking data: https://geerthofstede.com/research-and-vsm/dimension-data-matrix/*.

* *p < 0.05. n/d, no dimension available*.

## Discussion

Using constructs from Hofstede's Values Survey Module (VSM), we uniquely identified areas of cultural variability in a medical student and trainee population. It is important to remember, however, that not all students and practitioners within a single cluster are the same: we all have our own unique set of intersecting identities of who we are and what we value (9, 40), playing out in different ways within different contexts (9). Given this, we are mindful that care must be taken, as with any study, as we move through our discussion to contain our extrapolations accordingly. Thus, we consider that our findings will facilitate educators' conversations with a variety of stakeholders around cultural points of difference and similarities across medical students and trainees learning and working cross-culturally.

Focusing on our study population across different countries and cultural dimensions scores (RQ1), the construct of power shows the greatest homogeneity, followed by masculinity. We hypothesize these are areas that are likely to be the least problematic for cultural difference. Other research bears this out: for example, research suggests medical students across different cultures find challenging seniors in the face of professionalism dilemmas equally problematic (e.g., requests by seniors to participate in activities they feel to be unprofessional) (41–44). The construct of uncertainty shows the greatest diversity. This is an important finding, which we discuss further below (45, 46).

Regarding gender, two countries display a large disparity: notably Japan, with females scoring *high* and their male counterparts scoring *low* on indulgence (having the second largest variance). Although other research has found gender differences across Hofstede's dimensions in other countries (e.g., individualism, power, and orientation within Kuwaiti educators) (47), and gender differences in Japan for other dimensions power and individualism (males significantly higher) (14), this is the first study we know of to identify gender differences for indulgence. Our finding maps onto Hofstede's suggestion that males are socialized with “tougher” cultural values (greater restraint) than females [(9), p. 286]. Thus, we suggest gender is taken into account when considering the impact of learning and working in a culture different to one's own.

Country-by-gender profiles across cultural dimensions (RQ2) were meaningfully classified into four clusters, demonstrating complexity of variability across dimensions and traditional Eastern-Western cultures. Clusters 1 and 2 comprise predominately Western countries with the notable difference of Japanese females. Clusters 3 and 4 comprise predominately Eastern countries. Furthermore, with the exception of Australia, Japan, and South Africa, males and females from each country fall into the same cultural cluster. Particularly interesting is the patterning between clusters and cultural dimensions cutting across traditional Eastern-Western divides. For example, Clusters 1 and 4 are similarly high in power, but significantly different to Clusters 2 and 3 (displaying low scores). Thus, one hypothesis could be that students/trainees from a country in Cluster 2 (e.g., Chile) might find it hard to communicate with their seniors when working in a Cluster 1 culture (e.g., Ireland). Uncertainty has a more complex pattern, with significant differences between Clusters 1 (lowest) and 2 (mid-point in our data), which are both significantly different to Clusters 3 and 4 (high). We might hypothesize that this could account for some of the difficulties experienced by international students and trainees in their performance and integration (48–52).

For RQ3 (correlations between general and our study population data), notably masculinity and uncertainty are uncorrelated. Thus, health professions' education research relying on general rankings for their interpretations in these areas is likely to grossly misrepresent the very population they are studying. Differences between data sets might be due to a number of issues including shifts in cultural norms over time due to globalization and mobility or professional socialization (9, 10).

Uncertainty shows the most variance within our data, being different in our study population than the general population, with country-by-gender clustering having a high effect size. Further, uncertainty is central to medical students' and trainees' learning and working environments, and often overlooked (45). How individuals deal with uncertainty is a personal issue mediated through institutions (e.g., family, schooling, the state) (9). Uncertainty measures the extent to which members of a culture feel helpless or anxious by ambiguous situations. In medicine this might include limitations of current knowledge, patient management complexities and outcomes (53). In our data, China (F/M) Taiwan (F/M), Indonesia (F/M), Malaysia (F/M), Sri-Lanka (F), Korea (M), and Pakistan (F) form the high-uncertainty cluster, suggesting a general discomfort of the unknown. Both genders in Israel, Australia, Ireland, and New Zealand form the low-uncertainty group, suggesting a higher tolerance for ambiguity. Future research might focus on situations where students or practitioners need to make decisions in highly ambiguous contexts (e.g., ill-defined situations, the COVID-19 pandemic) to examine whether the cultural dimension of uncertainty is at play. Indeed, when working in a culture different to one's own, there might be various issues around reluctance to disclose uncertainty, the relative need for structure, (in)decisiveness, closed/open-mindedness, and avoidance of ambiguity (54). Physicians' low tolerance of ambiguity and uncertainty is associated with anxiety (55, 56), when the context is unsupportive (9, 10) or when experience is lacking (55). It may be worthwhile that educators initiate discussions around managing uncertainty around these issues in teams to mitigate undue stress (57). Furthermore, in educational settings, closer and more frequent supervision might be useful, in addition to allowing students and trainees to “sit” with their uncertainties in a supportive environment with seniors role-modelling being comfortable with uncertainty (56).

As with any study our work has limitations. Despite using Hofstede's statistical recommendations with countries sampled across multiple medical schools, we have an issue of sample representativeness. This means that we were unable to match participant demographics to medical school demographics for each country. However, non-representative, non-random, convenience, samples have been successfully used to compare nations for decades; comprising one of the most frequent examples of this type of research (9, 10, 18, 19). The theoretical basis for conducting a comparative study with such samples is the concept of *national context effects* (18, 19), and in this way we appeal to Firestone's analytic generalization through the use of theory (58). Further cross-cultural research utilizing Hofstede's questionnaire in medical education is needed to clarify the relative strength of our findings.

Despite limitations, our study has strengths. The main strength of our study is its' uniqueness in employing Hofstede's cultural dimensions with medical student and trainees across a large range of countries, allowing an insight into patterns of medical students' and trainees' cultural dimensions from which medical education researchers might draw upon for further research hypotheses. In doing so, we recommend researchers use data that is specific to the population studied rather than rely on assumptions drawn from general-population data. As such, in the absence of studies including Hofstede's questionnaire in their own study design, we see our work as being a more accurate source of information than Hofstede's IBM-based data for medical education researchers who wish to utilize Hofstede's theory in their work to make sense of their cross-cultural data. Furthermore, our findings might lead medical education researchers to hypothesize about cultural differences in a range of situations (including those described in this article), leading them to examine these empirically. Through such work we might develop more rigorous studies leading to a greater understanding of how culture impacts medical students and trainees' everyday learning and professional experiences in cultures other than their own.

## Data Availability Statement

The datasets presented in this article are not readily available because, due to the nature of this research, participants of this study did not agree for their data to be shared publicly. Further inquiries can be directed to the corresponding author.

## Ethics Statement

The studies involving human participants were reviewed and approved by University of Tasmania, Flinders University, Griffith University, Monash University, Pontificia Universidad Catolica de Chile, Peking University, The University of Hong Kong, Mahatma Gandhi Institute of Medical Sciences, All India Institute of Medical Science, Christian Medical College, Faculty of Medicine University of Indonesia, Tarumanagara University, University of Bengkulu, Udayana University, Royal College of Surgeons in Ireland (RCSI), Tel Aviv University, University of Limerick, Kyoto University School of Medical Sciences, Universiti Sains Malaysia, Universiti Putra Malaysia, The University of Auckland, Khyber Medical University, University of Lahore, Islamic International Medical College, University of Pretoria, Stellenbosch University, Korea University, College of Medicine, Ajoo University, Gacheon University, University of Kelaniya, Chang Gung Memorial Hospital, and Medical Education Research Center. The participants provided their written informed consent to participate in this study.

## Author Contributions

All authors have made substantial contributions to the conception or design of the work, or the acquisition, analysis, or interpretation of data, specifically: S-YY and LVM conceived the study and designed the work. LVM, S-YY, MC, JC, SC, LZ, Y-ML, OK-M, HN, KO, TP, AR, AS, DS, AW, LW, and MY collected the data and undertook interviews. S-YY and PC undertook the analyses. LVM linked all comments and wrote the first draft of the manuscript. LVM developed the first draft of reviewer responses, all other authors commented on responses and amended where appropriate prior to submission. All authors commented on the interpretation of the analysis according to their own culture, revised it critically for important intellectual content, gave approval for the version to be published, and agree to be accountable for all aspects of the work.

## Funding

We would like to acknowledge our funders, Ministry of Science and Technology, Taiwan (Grant No. MOST 106-2511-S-182-012-MY2).

## Conflict of Interest

The authors declare that the research was conducted in the absence of any commercial or financial relationships that could be construed as a potential conflict of interest. The reviewer JB declared a shared affiliation with one of the authors, LVM, to the handling editor at time of review.

## Publisher's Note

All claims expressed in this article are solely those of the authors and do not necessarily represent those of their affiliated organizations, or those of the publisher, the editors and the reviewers. Any product that may be evaluated in this article, or claim that may be made by its manufacturer, is not guaranteed or endorsed by the publisher.
