# Supplementary material for: Medical Students' and Trainees' Country-By-Gender Profiles: Hofstede's Cultural Dimensions Across Sixteen Diverse Countries
Source: Front Med (Lausanne). 2022 Feb 8;8:746288. doi: 10.3389/fmed.2021.746288 (PMC8862177; doi:10.3389/fmed.2021.746288)
Supplement: Supplementary file 1 [file Data_Sheet_1.docx]

Supplementary Table 1: Participants’ nationalities (n = 2529)

| **Nationality** | **Frequency** |
| --- | --- |
| Pakistani | 436 |
| Indonesian | 264 |
| Indian | 262 |
| Chinese | 235 |
| Taiwanese | 192 |
| Malaysian | 181 |
| Sri-Lankan | 111 |
| Australian | 106 |
| New Zealander | 101 |
| Israelian | 97 |
| South African | 95 |
| South Korean | 85 |
| Chilian | 83 |
| Irish | 57 |
| Japanese | 57 |
| Hongkongers | 41 |
| British | 25 |
| American | 22 |
| Canadian | 21 |
| Arabs | 8 |
| Singaporean | 6 |
| Afghan | 5 |
| Colombian | 4 |
| Ecuadorian | 3 |
| Bhutanese | 2 |
| Bolivians | 2 |
| Dutch (nationaliteit: Nederlands) | 2 |
| Persian | 2 |
| Bruneian | 1 |
| Cuba | 1 |
| Ecuadorian | 1 |
| Egyptian | 1 |
| Filipino | 1 |
| German | 1 |
| Grecian | 1 |
| Kuwaiti | 1 |
| Lithuanian | 1 |
| New Zealand European | 1 |
| Nigeria | 1 |
| Polish | 1 |
| Portuguese | 1 |
| Qatari | 1 |
| Saudi Arabia | 1 |
| Solomon Islander | 1 |
| Sudanese | 1 |
| Swazi | 1 |
| Taiwanese Malaysian dual citizen | 1 |
| Thai | 1 |
| Trinidad and Tobago | 1 |
| Ugandan | 1 |
| Yemeni | 1 |
| Zimbabwean | 1 |

Supplementary Table 2: Participated institutions

| **University/institution** | **Frequency** |
| --- | --- |
| Australia - Flinders University | 18 |
| Australia - Griffith University | 56 |
| Australia - Monash University | 47 |
| Australia - University of Tasmania | 24 |
| Canada - McGill University | 5 |
| Chile - Pontificia Universidad Católica de Chile | 86 |
| Chile - Universidad de Concepción | 6 |
| China - Beijing Hospital | 2 |
| China - Institute on Aging | 2 |
| China - Nanjing University | 2 |
| China - Peking Union Medical College | 4 |
| China - Peking University | 195 |
| China - Shenzhen District Hospital | 1 |
| China - Soochow University | 1 |
| China - Southern Medical University | 1 |
| China - Sun Yat-sen University | 2 |
| China - Tsinghua University | 1 |
| China - Union hospital | 1 |
| Croatia - Zagreb university | 1 |
| Hong Kong - The University of Hong Kong | 58 |
| Hungary - Szeged university | 1 |
| Hungary - University of Debrecen | 1 |
| India - All India Institute of Medical Science | 116 |
| India - Christian Medical College | 55 |
| India - Mahatma Gandhi Institute of Medical Sciences | 83 |
| India - NIMS Jaipur | 1 |
| Indonesia - Universitas Bengkulu | 76 |
| Indonesia - Universitas Indonesia | 114 |
| Indonesia - Universitas Tarumanagara | 43 |
| Indonesia - Universitas Udayana | 32 |
| Ireland - NUI Galway | 45 |
| Ireland - Royal College of Surgeons in Ireland (RCSI) | 31 |
| Ireland - University of Limerick (UL) | 17 |
| Israel - Bar Ilan | 13 |
| Israel - Ben Gurion University | 8 |
| Israel - Hebrew University | 22 |
| Israel - Technion | 10 |
| Israel - Tel Aviv University Sackler School of Medicine | 43 |
| Italy - University of bologna | 1 |
| Italy - University of Florence | 1 |
| Japan - Jichi Medical University | 23 |
| Japan - Kyushu University | 11 |
| Japan - Shinshu University | 9 |
| Japan - University of Tokyo | 3 |
| Japan- Kyoto University | 10 |
| Malaysia - Monash University | 59 |
| Malaysia - Universiti Putra Malaysia | 51 |
| Malaysia - Universiti Sains Malaysia | 77 |
| Netherlands - Vrije Universiteit Amsterdam | 1 |
| New Zealand - The University of Auckland | 63 |
| New Zealand - University of Otago | 59 |
| Pakistan - Khyber Medial University | 159 |
| Pakistan - Rehman medical college | 2 |
| Pakistan - Riphah International University | 59 |
| Pakistan - University of Lahore | 227 |
| Romania - Carol Davila University of Medicine and pharmacy | 2 |
| South Africa - Stellenbosch University | 80 |
| South Africa-University of South Africa | 1 |
| South Korea - Ajoo University | 28 |
| South Korea - Gacheon University | 22 |
| South Korea - Korea University College of Medicine | 33 |
| Sri-Lanka - Faculty of Medicine, University of Kelaniya | 36 |
| Sri-Lanka - University of Jayawardenapura | 19 |
| Sri-Lanka - University of Peradeniya | 53 |
| Taiwan - Chang Gung Memorial Hospital | 92 |
| Taiwan - Chang Gung University of Science and Technology | 38 |
| Taiwan - China Medical University | 1 |
| Taiwan - Chung Shan Medical University | 2 |
| Taiwan - Fu Jen Catholic University | 3 |
| Taiwan - Local clinic | 1 |
| Taiwan - Mackay Medical College | 8 |
| Taiwan - National Cheng Kung University | 8 |
| Taiwan - National Defense Medical Center | 2 |
| Taiwan - National Taiwan University Hospital | 1 |
| Taiwan - National Yang-Ming University | 22 |
| Taiwan - Taipei Medical University | 8 |
| Taiwan - Taiwan number 1 | 1 |
| Taiwan - Tzu Chi University | 16 |
| UK - Newcastle University | 10 |
| UK - SGUL University of Nicosia Medical School | 2 |
| UK - University College London | 1 |

Supplementary Table 3**:** Original ranking of country data for our study

|  | POWER | INDIVIDUALISM | MASCULINITY | UNCERTAINTY | ORIENTATION | INDULGENCE |
| --- | --- | --- | --- | --- | --- | --- |
| Australia | 9 | 3 | 14 | 15 | 10 | 5 |
| Chile | 7 | 5 | 15 | 12 | 4 | 1 |
| China | 3 | 15 | 3 | 3 | 14 | 16 |
| Hong Kong | 8 | 13 | 4 | 11 | 1 | 11 |
| India | 5 | 14 | 5 | 7 | 9 | 13 |
| Indonesia | 6 | 12 | 11 | 2 | 3 | 6 |
| Ireland | 10 | 4 | 10 | 14 | 16 | 2 |
| Israel | 15 | 2 | 1 | 16 | 13 | 3 |
| Japan | 2 | 6 | 16 | 9 | 2 | 8 |
| Malaysia | 1 | 10 | 8 | 4 | 6 | 12 |
| New Zealand | 12 | 1 | 9 | 13 | 15 | 4 |
| Pakistan | 4 | 16 | 2 | 6 | 12 | 15 |
| South Africa | 13 | 7 | 6 | 10 | 8 | 7 |
| South Korea | 11 | 8 | 12 | 8 | 5 | 9 |
| Taiwan | 16 | 9 | 13 | 5 | 7 | 10 |
| Australia | 14 | 11 | 7 | 1 | 11 | 14 |


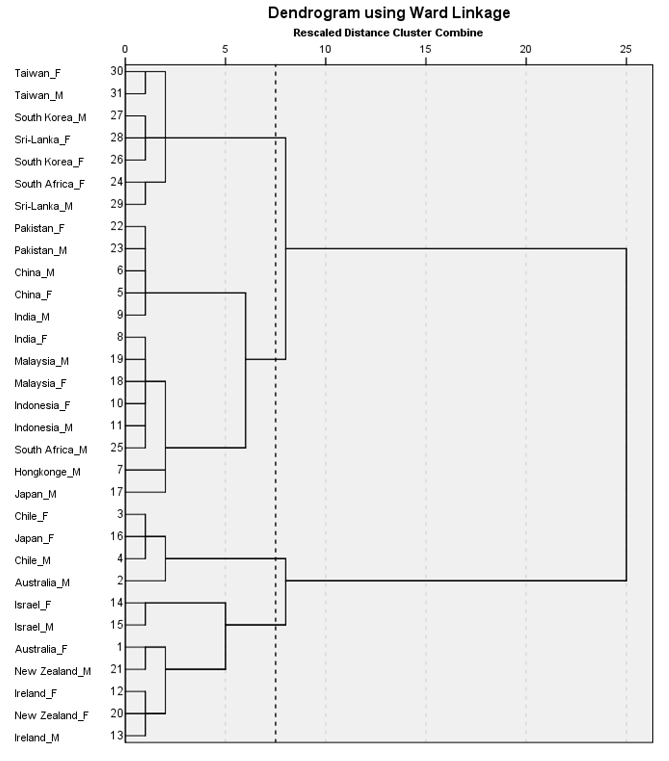


Supplementary Figure 1**:** Hierarchical tree of the ward algorithm method by country and gender. The dotted line indicates the optimal stopping location, and four distinct clusters are identified: M = male and F = female. Note: In the two-cluster model, cluster 1 and cluster 2 were grouped together as one cluster, and clusters 3 and 4 were grouped together as another cluster. The four-cluster model showed statistically significant results for all six dimensions, while the two-cluster model did not: the values of POWER *t*(29) = .55, *p* > .05 and MASCULINITY *t*(29) = .35, *p* > .05 showed no significant differences between two different clusters.
